# Granulomatosis with Polyangiitis in Adolescence: Two Distinct Presentations

**DOI:** 10.1155/2021/6642910

**Published:** 2021-06-19

**Authors:** Rafael Figueiredo, Inês Pires Duro, António Marinho, Conceição Mota, Margarida Guedes, Carla Zilhão

**Affiliations:** ^1^Department of Pediatrics, Centro Materno-Infantil do Norte–Centro Hospitalar Universitário do Porto, Porto, Portugal; ^2^Department of Internal Medicine, Hospital de Santo António–Centro Hospitalar Universitário do Porto, Porto, Portugal; ^3^Unit of Pediatric Nephrology, Department of Pediatrics, Centro Materno-Infantil do Norte–Centro Hospitalar Universitário do Porto, Porto, Portugal; ^4^Unit of Pediatric Rheumatology, Department of Pediatrics, Centro Materno-Infantil do Norte–Centro Hospitalar Universitário do Porto, Porto, Portugal

## Abstract

*Introduction*. Granulomatosis with polyangiitis (GPA) is a rare disease in pediatric age. We report two cases with distinct presentations. *Case Reports*. A seventeen-year-old male with prolonged febrile syndrome, cough, and constitutional symptoms. CT-scan showed cavitated lesions of the lung and bronchial biopsy a necrotizing inflammatory process. The remaining investigation revealed hematoproteinuria and positive C-ANCA and anti-PR3. Complications: Bilateral acute pulmonary thromboembolism, splenic infarction, and extensive popliteal and superficial femoral deep vein thrombosis. He was treated with corticosteroids, immunoglobulin, rituximab, and anticoagulation. Rituximab was maintained every six months during the first two years. Control angio-CT was performed with almost complete resolution of previous findings. In a twelve-year-old female with inflammatory signs of the limbs, investigation showed myositis of the thigh and tenosynovitis of the wrist, normocytic normochromic anemia (Hg 9.4 g/dL), mild elevation of inflammatory markers, and high creatine kinase. During hospitalization, she presented an extensive alveolar hemorrhage associated with severe anemia and positive C-ANCA and anti-PR3. Clinical deterioration prompted intravenous methylprednisolone pulses and plasmapheresis. Induction therapy with rituximab and prednisolone showed good results. Rituximab was maintained every six months, for 18 months, with gradual tapering of corticoids. *Discussion*. GPA is a systemic disease with variable clinical presentation and severity. Pediatric patients have similar clinical manifestations to adults but different frequencies of organ involvement; constitutional symptoms are also more common. We highlight the different presentation of these two cases, as well as the need for an individualized approach. Rituximab has been used for both induction-remission and maintenance therapy, with good results, particularly in young patients.

## 1. Introduction

Granulomatosis with polyangiitis (GPA), formerly known as Wegener's granulomatosis, is an infrequent systemic disease, necrotizing vasculitis, which predominantly involves small-sized vessels [1, 2]. It is a rare disease in pediatric age, with an unknown juvenile onset incidence, though ranging from 0.5 to 8 cases/million per year with a predominance in adolescent females [1–4].

GPA is associated with the presence of antineutrophil cytoplasmic antibodies (ANCA), the majority (90%) being PR3-ANCA, although in some patients, MPO-ANCA can be found as well.

Their classification is based upon the clinical phenotype, size of the involved blood vessel, histopathology of the damaged vessel, or the presumed underlying disease pathogenesis. Regarding the GPA diagnostic criteria, a pediatric age adjustment of the American College of Rheumatology (ACR) criteria was approved in 2010, the EULAR/PRINTO/PRES criteria [5].

The authors present two cases of GPA PR3-ANCA in adolescent patients with severe but distinct clinical presentations.

## 2. Case Presentation

**Case 1 .**

**Figure 1:**
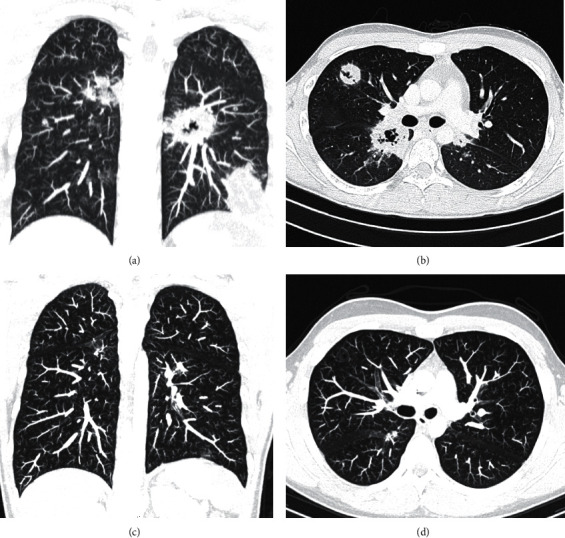
CT scan showing parenchymal nodular areas with internal cavitation (a, b) with almost complete resolution after 4 months (c, d), Case 1.

A previously healthy 17-year-old Caucasian male patient, with no family history of known rheumatic diseases, was observed in the emergency department with a history of fever, dry cough, and pleuritic chest pain for the past 24 h. Chest X-ray revealed an hypotransparency on the left inferior lobe, and antibiotic therapy was initiated, based on the suspicion of community-acquired pneumonia. Five days later, the symptoms worsened, with the onset of conjunctival hyperemia, odynophagia, asthenia, anorexia, and nocturnal diaphoresis. In the emergency department, his weight was 70 kg (had decreased 4 kg compared to two months earlier), and physical examination revealed an axillar temperature of 37.6°C, bilateral bulbar conjunctival hyperemia, and bibasal decreased breath sounds. He was normotensive, and there was no identifiable organomegaly or adenopathy. Laboratory testing revealed hemoglobin 12.3 g/dL (MCV 80 fL, RDW 12.4%), lymphopenia (950/*μ*L), increased DHL (498 U/L), and increased inflammatory markers (reactive C-protein 106.5 mg/L and erythrocyte sedimentation rate 71 mm/h). Chest X-ray exposed multiple nodular hypotransparent lesions scattered by both the lungs, and a CT scan showed parenchymal nodular areas with internal cavitation (Figures 1(a) and 1(b)). Faced with the possibility of a necrotizing pneumonia, he was admitted in the pediatric department and started empirical therapy with ceftriaxone and vancomycin. However, fever persisted, and eight days later, he started complaining of diffuse abdominal pain. A new thoracoabdominal CT scan presented worsening pulmonary lesions and an enlarged spleen (13 cm larger diameter), with marked heterogenicity. Renal function was normal (plasma creatinine 0.8 mg/dL, urea 25 mg/dL), and urinalysis presented a protein count of 25 mg/dL, with 10–25 erythrocytes per high resolution field. He underwent a bronchofibroscopy, which showed a lesion in the left portion of the carina and whose biopsy revealed a necrotizing inflammatory process. An autoimmune workup showed a positive C-ANCA (1/640) and anti-PR3 (2250 UQ) with negative antinuclear antibodies (ANAs), anti-double-stranded DNA (dsDNA), and antimyeloperoxidase (MPO) and a normal immunoglobulin and complement assessment. Considering a systemic ANCA-positive vasculitis, he started prednisolone 60 mg/day. Due to an extensive popliteal deep venous thrombosis and a superficial femoral thrombosis, anticoagulation therapy was also instituted. Five days later, an acute pulmonary thromboembolism and a splenic infarction were diagnosed, leading to intravenous methylprednisolone pulses (1 g for 3 days) and immunoglobulin perfusion (2 cycles of 0.4 g/kg, 5 days). To exclude pulmonary tuberculosis, a cultural and PCR search for BK was carried out. After negative results, rituximab was initiated (375 mg/m^2^/week, 4 weeks). *Pneumocystis jirovecii* prophylaxis with cotrimoxazole was also introduced. An episode of macroscopic hematuria was also noticed that resolved after 24 h. Cardiac evaluation revealed a noncircumferential pericardial effusion, without hemodynamic compromise. Ophthalmologic and ear-nose-throat (ENT) evaluations were normal. During outpatient follow-up, an angio-CT scan revealed complete resolution of pulmonary thromboembolism and nearly complete resolution of cavitated granulomas 4 months after onset of the disease (Figures 1(c) and 1(d)), and at this moment, clinical remission was achieved. The anticoagulation treatment was suspended after 9 months, with a progressive tapering of oral glucocorticoid and suspension after 12 months. He underwent maintenance therapy with rituximab every 6 months during the first 2 years. Despite hypogammaglobulinemia (minimum of 496 mg/dL), no infections or complications were reported, and immunoglobulin replacement therapy was not required. During the 43 months of follow-up, there is a reference to mild sinusitis symptoms with no further complaints. Pulmonary function tests and renal function remained normal. Regular analytical monitoring demonstrated normalization of ESR and a progressive decrease of C-ANCA titers.

**Case 2 .**

**Figure 2:**
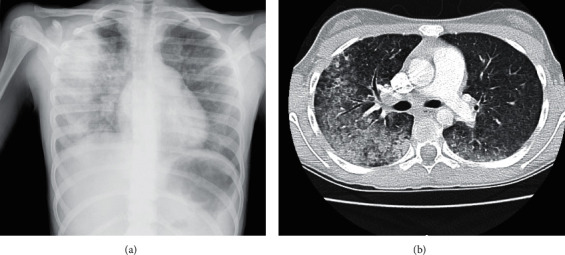
Chest X-ray (a) and CT scan (b) showing signs of alveolar hemorrhage, Case 2.

A 12-year-old Caucasian female with a history of asthma, with no familial history of known rheumatic diseases, was admitted in the emergency department with inflammatory signs of the posterior segment of the thigh and right wrist lasting the previous 10 days and sporadic complaints of lower limb myalgia lasting several months. Fever, having started that day, was also present. At physical examination, she was apyrexial, hemodynamically stable, normotensive, but pale. She weighed 35 kg, and her height was 146 cm. Cardiac evaluation revealed a systolic murmur II/VI (aortic focus), and inflammatory signs on the infragluteal region of the right thigh (10 cm of length) and on the right wrist (2-3 cm in diameter) were present. Ultrasound suggested myositis of the thigh and tenosynovitis of the wrist, and laboratory testing demonstrated a normocytic normochromic anemia (Hg 9.4 g/dL), mild leukocytosis (14360/*μ*L) with neutrophilia (10430/*μ*L), ESR 85 mm/h, mildly elevated C-reactive protein (53 mg/L), and an elevated creatine kinase (1423 U/L). She was then admitted in the pediatric department for investigation. During hospitalization, the mucocutaneous pallor intensified and hemoglobin dropped to 6.8 g/dL. During the following day, she started to show hemoptysis, dyspnea, and signs of respiratory distress, with increasing needs for supplementary oxygen. Chest X-ray and CT scan revealed signs of alveolar hemorrhage (Figure 2), and significant hematoproteinuria was detected (urine protein 0.4 g/g creatinine, erythrocytes 5–10 per field). Therefore, due to the presence of a pulmonary-renal syndrome, a diagnosis of a vasculitis was considered. An autoimmune workup revealed positive c-ANCA (1/640 titers), anti-PR3 (3285.3 UQ/), and antinuclear (IF) (1/80 mottled pattern) and negative anti-dsDNA, anti-MPO, myositis-specific, and myositis-associated autoantibodies. Immunoglobulin assay, leukocyte immunophenotyping, and complement assessment were normal.

Due to clinical deterioration, she was transferred to the pediatric intensive-care unit and initiated high-dose intravenous methylprednisolone pulse therapy (30 mg/kg), along with plasmapheresis. Inotropic support was required for 2 days, as well as transfusional support and mechanical ventilation (maximum fiO_2_ 31%) for 9 days. A total of 7 sessions of plasmapheresis were completed. Rituximab (375 mg/m^2^/week, 4 weeks) and oral prednisolone (40 mg/day) were chosen for induction therapy, and *Pneumocystis jirovecii* prophylaxis with cotrimoxazole was started. There was a progressive control of pulmonary hemorrhage and hematoproteinuria, with preserved renal function. Despite reference to self-limited episodes of epistaxis, ENT evaluation was normal. Ophthalmologic evaluation did not show any signs of uveitis or other lesions. During outpatient follow-up, she completed 4 weeks of rituximab with a gradual tapering of glucocorticoids to 5 mg/day (prednisolone) and cycles of rituximab every 6 months, for 18 months. Despite the absence of reported infections, she maintained a persistent hypogammaglobulinemia (minimum 469 mg/dL), and immunoglobulin replacement therapy was administered. Pulmonary function tests showed a mild obstructive pattern that improved over time.

Clinical remission was achieved 1 month after the onset of the disease, and no other significant complications were reported during the 43 months of follow-up.

## 3. Discussion

GPA is a systemic disease with variable severity and clinical presentations. Pediatric patients have clinical manifestations similar to adults, but with different frequencies of organ involvement [6]. These 2 cases demonstrate some of these differences. The most common clinical manifestations of childhood GPA at disease onset are related to upper airway involvement (82%), nephropathy (65%), lower respiratory tract disease (61%), and musculoskeletal (55%). Nonspecific systemic symptoms such as fever and fatigue are also frequent (73%) [4, 7].  Case 2 investigation was initially prompted by nonspecific musculoskeletal symptoms, despite that other findings were quickly found.  Case 1 presentation was pneumonia-like, with systemic and respiratory symptoms. GPA severity of lung involvement is variable, ranging from asymptomatic pulmonary lesions, nodular lesions, or cavities to diffuse alveolar hemorrhage that can be fulminant and dramatically life threatening, as presented in Case 2 [7]. On the other hand, it is also important to recall that GPA is associated with a significative increased risk of thromboembolic events, such as pulmonary embolism and deep venous thrombosis, at disease presentation and during follow-up, as described in Case 1 [7, 8]. Active disease seems to present a major risk factor, and despite the pathogenetic background being poorly understood, it appears to be related to endothelial function and integrity, induction of a state of hypercoagulability resulting from changes in pro- and anticoagulant factors, associated with an inflammation status and eventually the use of cyclophosphamide and high doses of glucocorticoids [8–11].

At diagnosis, antineutrophil cytoplasmic antibodies (ANCA) play an essential role. They form a heterogeneous group of antibodies that target antigens present mostly in azurophilic granules of polymorphonuclear leukocytes. Since the connection between ANCA and GPA was established, they have become part of routine diagnostic procedures. There are two main targets identified as enzymes, proteinase 3 (PR3), mainly associated with GPA, and myeloperoxidase (MPO), more frequently associated with microscopic polyangiitis. The distinction between GPA and other disorders is a common clinical problem, as several infections, such as tuberculosis and bacterial endocarditis, cystic fibrosis, and cocaine abusers, can have similar presentations and ANCA-positive titers [12].

Both described cases were PR3-ANCA positive with no other positive autoantibodies, a typical presentation of GPA. Both presented 3 EULAR/PRINTO/PRES GPA criteria, involving lung, renal, and PR3-ANCA titers, which made the diagnosis [5].  Case 2 also reported upper airway involvement with sporadic episodes of epistaxis, but with no other major complications.

Despite proper treatment, they both progressed to severe illness. In Case 1, we highlight the severe pulmonary involvement and multiple thromboembolic events requiring anticoagulation therapy; in Case 2, the massive alveolar hemorrhage required intensive-care ventilatory and ionotropic support and plasmapheresis. Renal involvement was present in both cases, though mild, transitory, and with preserved renal function.

In the absence of treatment, GPA can be life threatening and mortality can reach 80–93% in the first 2 years, due to disease progression or other complications. New staged therapeutic strategies have dramatically improved overall survival over the last decades [10, 13–15]. The most frequent induction treatment is the use of high-dose glucocorticoids in combination with cyclophosphamide (Cyp), followed by a lower dose of glucocorticoids and continued administration of immunosuppressive therapy [16]. The introduction of oral cyclophosphamide combined with high-dose glucocorticoids led to a substantial improvement in patient survival. Unfortunately, Cyp is associated with a high risk of toxicity, particularly at young ages, and may have serious side effects such as infertility, opportunistic infections, hemorrhagic cystitis, medullar aplasia, and risk of malignancy [16]. Intravenously Cyp reduces cumulative exposure and seems to be associated with fewer side effects, such as leukopenia and neutropenia, compared to oral Cyp. Nevertheless, there is also a considerable number of Cyp nonresponders or frequent relapses and progression of the disease [17–19]. Rituximab, a monoclonal antibody that targets B cells, has been used in both induction-remission and maintenance therapy, particularly in young patients, with good results in comparison to cyclophosphamide, as shown in RAVE and RITUXVAS Trials [20–22]. The former stated the noninferiority of rituximab therapy versus daily cyclophosphamide treatment for induction of remission in severe ANCA-associated vasculitis. The RAVE 18-month extension published 3 years later showed no significant differences at relapse, remission, or adverse event rates between the groups (treated with rituximab vs. Cyp) throughout the follow-up period. The RITUXVAS trial results, comparing rituximab versus Cyp in ANCA-associated renal vasculitis, presented a higher rate of ANCA negativity than that observed with Cyp regimens in previous studies. Also, rituximab does not impair fertility and carries an apparently lower malignancy risk. This is why, despite the lack of solid data in children, its use in the pediatric population has substantially increased [23]. In the reported cases, rituximab (375 mg/m^2^ body surface area, weekly for 4 weeks) plus high-dose glucocorticoids were used to induce remission, and due to disease severity, intravenous pulsed methylprednisolone was preferred to oral prednisolone in the first days of treatment.

Adjuvant therapies such as plasmapheresis may be beneficial in patients with identified renal disease or severe diffuse alveolar hemorrhage, according to the latest EULAR recommendations [16]. In selected patients with rapidly progressive renal disease, plasmapheresis appears to prevent end-stage kidney disease or death at 3 months, but without long-term benefits. Recently, the PEXIVAS trial (2020) demonstrated that the use of plasma exchange did not reduce the incidence of death or end-stage kidney disease [24, 25].

Therapy with rituximab is associated with increased risk for opportunistic infections, such as *Pneumocystis jirovecii* pneumonia, with significant morbidity and even mortality. Rituximab causes depletion of B lymphocytes which alter the function of T lymphocytes and may also cause hypogammaglobulinemia and failure of naive B lymphocytes to differentiate into plasma cells [26, 27]. For these reasons, it is widely recommended to maintain *Pneumocystis jirovecii* prophylaxis for at least the duration of B lymphocyte depletion following rituximab infusions in patients with GPA [16, 26].

Regarding maintenance therapy, latest EULAR recommendations indorse treatment with a combination of low-dose glucocorticoids and either azathioprine, rituximab, methotrexate, or mycophenolate mofetil. The MAINRITSAN trial compared low-dose rituximab every 6 months to a tapering dose of azathioprine for remission maintenance after induction with pulsed cyclophosphamide and demonstrated superiority of rituximab over daily oral azathioprine, particularly in patients PR3-ANCA positive [20]. The 6-month period was based on reported B-cell reconstitution and relapses after a median of 1 year in early studies of patients given rituximab for induction [28, 29]. Recent published results of the RITAZAREM trial, following induction of remission with rituximab, also demonstrated the superiority of rituximab over azathioprine for preventing disease relapse in patients with AAV with a prior history of relapse [30].

In both described cases, maintenance therapy consisted of rituximab every 6 months for 18–24 months, with good outcomes.

Close follow-up is mandatory, as the possibility of relapse is considerable, even in previously unaffected organ systems. ANCA titers monitorization can be helpful, although this is not consensual. Low titers seem to be associated with clinical remission, and negative titers after therapy are indicative of low risk for relapsing. On the other hand, some authors state that ANCA titers are not useful to predict relapse and should not be used alone to initiate prophylactic therapy [31, 32]. Nevertheless, when B cells are undetectable and ANCA remain negative, relapses are rare [33]. The MAINRITSAN2 trial, undertaken to evaluate ANCA and circulating CD19 + B cells as indicators to reinfuse rituximab to maintain remission, compared an individually tailored rituximab regimen with fixed-schedule rituximab infusions. Its results demonstrated a nonassociation of ANCA and CD19 + B cell monitoring with relapse, but its monitorization presented the benefit of aiding in the decision to reinfuse, leading to fewer rituximab infusions in the tailored-infusion arm [34]. In our cases, a fixed schedule of rituximab 500 mg every 6 months was used, with no relapses reported during the previously mentioned follow-up period.

As stated before, rituximab depletes CD20-expressing B cells, an effect that can persist for 6–12 months. This can cause hypogammaglobulinemia, characterized by reduction in circulating immunoglobulins, primarily immunoglobulin G, and can be persistent. Prolonged hypogammaglobulinemia predisposes to severe and recurrent infections, another reason why it is important to follow-up patients under rituximab therapy, with evaluation of immunoglobulin levels before and after rituximab administration [35, 36]. Severe hypogammaglobulinemia may require immunoglobulin replacement therapy, although specific cutoff IgG levels demanding immunoglobulin replacement therapy are unclear [32, 37]. Our two patients developed persistent hypogammaglobulinemia, but only Case 2 underwent replacement therapy. No relevant infection was reported in any of them.

Over the last few years, despite having been conducted numerous studies addressing the use of immunosuppressive medications in GPA, studies guiding use of glucocorticoids after the induction of remission have been lacking. Whether low-dose glucocorticoid contributes to maintaining the remission is still a matter of debate. Some authors support the use of long-term low-dose glucocorticoid, while others defend that it is ineffective at reducing relapses and exposes patients to the potential toxicity of high cumulative doses of glucocorticoid [38]. The results of a meta-analysis that explored the effect of different glucocorticoid regimens on relapse rates among patients with GPA suggest that early withdrawal is associated with more relapses and that a low-dose glucocorticoid for greater than 12 months would provide a noticeable benefit. However, they suggest that these benefits may be restricted to patients at high risk of a relapse [38]. Nonetheless, given the possibility of significant differences between short- and long-term protocols for altering disease activity, more quality studies are mandatory to address this question. Regarding our patients, only Case 2 maintained a low-dose glucocorticoid regimen after rituximab.

## 4. Conclusions

Although rare in pediatric age, the knowledge of GPA manifestations will allow an earlier diagnosis and a more targeted approach. Timely remission induction treatment is mandatory to reduce disease high rates of morbidity and mortality. In recent years, new therapeutic options increased the survival rate, minimizing adverse effects of treatment. As presented, glucocorticoids and rituximab were used with good outcomes and few side effects, allowing the patients to be in remission after 43 months of follow-up. Pediatric studies are essential since most of the data are extrapolated from adult studies.
